# Trans-Mediated, Cis-Inhibited Paradoxal Activity of Clostridium perfringens Enterotoxin (c-CPE) in Modulating Epithelial Permeability

**DOI:** 10.1021/acs.molpharmaceut.4c01205

**Published:** 2025-03-11

**Authors:** Julieta M. Sanchez, Marianna T. P. Favaro, Hèctor López-Laguna, Eloi Parladé, Angela Di Somma, Isolda Casanova, Ugutz Unzueta, Ramón Mangues, Esther Vazquez, Eric Voltà-Durán, Antonio Villaverde

**Affiliations:** † Institut de Biotecnologia i de Biomedicina (IBB), Universitat Autònoma de Barcelona, Barcelona 08193, Spain; ‡ Centro de Investigación Biomédica en Red de Bioingeniería, Biomateriales y Nanomedicina, Instituto de Salud Carlos III, Barcelona 08034, Spain; § Departamento de Química, Cátedra de Química Biológica, Facultad de Ciencias Exactas, Físicas y Naturales, ICTA, Universidad Nacional de Córdoba, Av. Vélez Sársfield 1611, Córdoba 5016, Argentina; ∥ Instituto de Investigaciones Biológicas y Tecnológicas (IIByT), CONICET-Universidad Nacional de Córdoba, Córdoba 5016, Argentina; ⊥ Departament de Genètica i de Microbiologia, Universitat Autònoma de Barcelona, Barcelona 08193, Spain; # Department of Chemical Sciences, University of Naples “Federico II”, Vicinale Cupa Cintia 26, Naples 80126, Italy; ∇ Institut de Recerca Sant Pau (IR SANT PAU), Barcelona 08041, Spain; ○ Josep Carreras Leukaemia Research Institute (IJC), 08916 Badalona, Spain

**Keywords:** recombinant protein, nanoparticles, drug delivery, claudin, cCPE, transactivity

## Abstract

In the context of transdermal delivery, favoring the drug permeability of epithelia through convenient formulations would open new opportunities for local versus systemic drug delivery, envisaging higher patient comfort and an enhanced therapeutic effect. Ligands of tight junctions are interesting agents that enhance epithelial permeability by relaxing the protein complexes that form them. The C-terminal domain of Clostridium perfringens enterotoxin (c-CPE), which binds claudins, one of the tight junction (TJ) components, has been explored here as a functional domain in modular recombinant proteins, to evaluate its ability to self-promote its paracellular epithelial passage in a Caco-2 cell monolayer model. c-CPE-containing fusion proteins bind cells in the absence of internalization and cytotoxicity and support the passage, in trans, of other fusion proteins devoid of c-CPE. However, c-CPE-carrying proteins fail to cross the epithelia by themselves, probably because their affinity for TJs immobilizes them in the intercellular space. Therefore, while recombinant c-CPE versions have been here confirmed as convenient epithelial-permeabilizing agents, a paradoxical behavior has been observed where this effect is only successful when applied in trans, specifically on entities that lack c-CPE. Then, c-CPE itself inhibits the paracellular mobility of carrier molecules, not being suited as a self-driver (in c-CPE-drug complexes) for drug delivery through epithelia.

## Graphical abstract

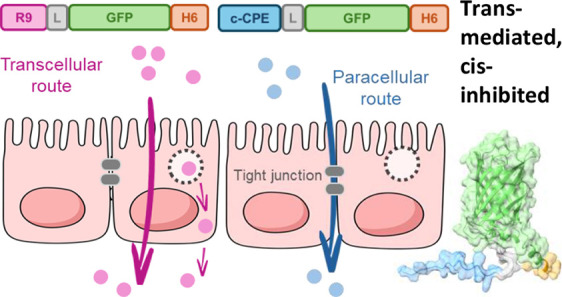

## Introduction

Enhancing the molecular permeability of biological barriers, such as skin or gut epithelia, is a main challenge in drug delivery,^1–3^ because successful approaches in this regard might favor local delivery, increase drug efficacy, and improve patient comfort. Epithelial cells are attached to each other by the junction complex, in which tight junctions (TJs) are critical for the barrier function.^4^ TJs are composed of a set of different families of cross-interacting transmembrane proteins (including Claudins, Occludin, JAMs, Tricellulin, MarvelD3) and cytoplasmic scaffold proteins (such as ZO-1, ZO-2, ZO-3, Cingulin, Paracingulin, and Afadin),^5,6^ forming complexes that regulate the passage of ions, water, and other molecules across the cell layer while keeping its integrity and function. Aiming at drug delivery issues, TJ performance has been often manipulated by claudin-binding proteins,^7^ as well as by signaling molecules like PKC, that modify the function of tight junctions through phosphorylation.^8^ The enterotoxin from C. perfringens (CPE), a main cause for food poisoning,^9^ disrupts the TJs, leading to increased membrane permeability by pore formation and consequent cell death.^10^ Claudin-3 and claudin-4 are high-affinity receptors for CPE, while claudin-1 and claudin-2 exhibit weaker interactions. The binding affinity of CPE to claudin-3 shows an association constant (Ka) of approximately 1.0 × 10^8^ M^–1^.^11^ The carboxy-terminal domain of this protein (c-CPE), devoid of cytotoxicity, also targets claudin-3 and claudin-4, although its specificity can be modulated by genetic engineering.^12^ This protein segment has been tested as a probe or diagnostic tool^13^ and as a modulator of the TJ performance,^14^ while the whole CPE protein has been explored as an antitumoral cytotoxic drug for claudin-overexpressing cancers, such as thyroid and lung cancers.^15^ c-CPE can be easily produced in recombinant fusion proteins,^13^ which makes this domain a convenient ligand for targeting cancer tissues. This allows envisaging selective drug delivery of the whole CPE^16,17^ or attached or combined chemicals such as Taxol,^17^ or proteins such as TNF.^18^ In this regard, many studies propose engineering or enhancing the selectivity for the specific claudins that are relevant in cancer^19–22^ or antiviral^23^ therapies. Despite its clinical interest, much less has been explored about how c-CPE might assist in transepithelial drug delivery through TJ relaxation. In this context, it has been shown, in in vitro cell coculture models, that this peptide alone activates the paracellular pathway for the transepithelial passage of chemicals such as fluorescein and large proteins such as albumin,^24^ in a reversible, nontoxic fashion,^25^ because c-CPE does not cause any permanent damage on the TJs.^24^ The TJ relaxation mediated by this peptide is related to its ability to bind claudin 3 and claudin 4, which weakens the homophilic interaction of the TJ strands from contiguous cells.^26^ The loss of the natural TJ barrier function^25–27^ is what allows the flow of molecules, enabling c-CPE and similar peptides as potential permeabilizers. Among other approaches, the use of c-CPE mutants has allowed identifying cell protein kinases involved in the TJ relaxation process.^28^

A deeper understanding of how the c-CPE protein domain might be utilized as a drug delivery enhancer would indeed assist in the design of functional transepithelial delivery systems in such a therapeutic scenario. Also, it would be interesting to examine if c-CPE, as part of modular large multifunctional polypeptides, might keep its properties as an enhancer of epithelial permeability. In this context, we have explored here the mechanics of the CPE-based enhanced epithelial penetrability using a Caco-2-based in vitro epithelial model, which has rendered useful insights regarding the trans- but also the cis-activity of c-CPE, being part of complex fusion proteins, as a modulator of TJs.

## Materials and Methods

### Protein Production and Purification

Genes were provided by Geneart (Thermo Fisher) already subcloned into a pET22b plasmid (Novagen). These plasmids were transformed into E. coli BL21 (DE3) (Novagen), and the recombinant proteins were produced overnight at 20 °C upon induction with 0.1 mM isopropyl- β-D-1-thiogalactopyranoside (IPTG), except for c-CPE-FGF2-H6, which was produced in E. coli BL21 (pLys) using 1 mM IPTG. After induction of gene expression, the bacterial pellet was collected and resuspended in wash buffer (20 mM Tris-HCl, 500 mM NaCl, 10 mM Imidazole, pH 8) in the presence of protease inhibitors (cOmplete EDTA free, Roche Diagnostics) and disrupted by two rounds of sonication (amplitude 40%, pulse on/off: 1, 3 s). The soluble fraction was separated by centrifugation at 15,000 *g* for 45 min at 4 °C and then filtered (0.22 μm) before proceeding to protein purification by immobilized metal affinity chromatography (IMAC) in 5 mL HisTrap HP columns (Cytiva) in an Äkta Pure system (Cytiva). Proteins were eluted by a linear gradient of imidazole in an elution buffer (20 mM Tris-HCl, 500 mM NaCl, 500 mM imidazole, pH 8), followed by dialysis cycles for complete imidazole removal. GFP-H6 was dialyzed against phosphate buffer (7.5 mM Na_2_HPO_4_, 2.5 mM NaH_2_PO_4_ pH 7.4), c-CPE-GFP-H6 was dialyzed against phosphate buffer with 500 mM NaCl pH 7.4 and R9-GFP-H6 against phosphate buffer with 333 mM NaCl pH 7.4. The protein c-CPE-FGF2-H6 was dialyzed against sodium carbonate and salt solution (166 mM NaCO3H, 333 mM NaCl, pH 8). Protein purity was verified by SDS-PAGE electrophoresis in a TGX stain-free gel (Bio-Rad). Protein concentration was determined by Bradford Assay (Bio-Rad Protein Assay Dye Reagent Concentrate, Bio-Rad).

### Protein Modeling and Visualization

ColabFold v1.5.5 (AlphaFold2 using MMseqs2) was used to generate the predicted three-dimensional models of GFP-H6, R9-GFP-H6, c-CPE-GFP-H6, and c-CPE-FGF2-H6.^29^ Generated models were represented by UCSF ChimeraX 1.7.1.,^30^ using electrostatic molecule display to represent charge distribution and b-factor to display confidence of the manually superimposed models. Net charge of the proteins and peptides at pH 7.4 was calculated using the Prot pi Web server v2.2.29.

### Specific Fluorescence

GFP fluorescence emission spectra were determined for each protein at 0.5 mg/mL in a Cary Eclipse spectrofluorometer (Agilent Technologies) with a quartz cell with a path length of 2 mm. The excitation slit was set at 2.5 nm, and the emission slit was set at 5 nm. The excitation wavelength (λ_ex_) used was 488 nm. The specific fluorescence was comparatively calculated as the fluorescence intensity at 512 nm (arbitrary units) relative to a concentration of 1 mg/mL. Percentual values for each protein were calculated (considering 100% specific fluorescence of GFP-H6).

### Circular Dichroism

Circular dichroism spectra were acquired with a JASCO J-715 spectropolarimeter (JASCO, Oklahoma City, OK, USA) applying a 0.2 mm path length quartz cell. Each spectrum corresponds to an average of five scans. The scan speed was set at 100 nm min^–1^ with a 1 s response time. Measurements were obtained as ellipticity (θ) in millidegrees (mdeg) in the 200–260 nm range.

### Dynamic Light Scattering

Volume size distribution of proteins was determined by dynamic light scattering (DLS) at 633 nm and 25 °C in a Zetasizer Advance Pro (Malvern Instruments), measured in five replicates. For protein stability analysis, the size measurement was carried out in a ZEN2112 3 mm quartz batch cuvette using temperatures of 20, 37, 50, 60, 70, 80, and 90 °C.

### Protein Internalization and Cell Viability Assay

HeLa cells (ATCC, CCL-2) were routinely cultured in Minimum Essential Medium (MEM Alpha Medium 1X + GlutaMAX) (Gibco) supplemented with 10% fetal bovine serum (Gibco) in a humidified atmosphere with 5% CO_2_ at 37 °C. For internalization assays, HeLa cells were cultured in 24-well plates in their medium until 70% confluence was reached. The medium was then exchanged for OptiPRO Serum Free Medium before the addition of proteins. Protein uptake was determined at 1 and 24 h at a final concentration of 1 μM. Cells were detached, and externally bound protein was removed with Trypsin-EDTA at 1 mg/mL exposure for 15 min at 37 °C. Intracellular protein fluorescence was determined by flow cytometry using a CytoFLEX (Beckman Coulter) with blue laser (488 nm). The intracellular fluorescence values for each condition were divided by the background fluorescence values of control cells in the same experiment, resulting in the relative fluorescence values expressed in the graph (times of fluorescence higher than that of unexposed cells). Experiments were performed four times and expressed as relative fluorescence values ± standard error.

For cell viability assays, HeLa cells were grown as previously described and transferred to opaque-walled 96-well plates to a final concentration of 3,500 cells per well and grown for 24 h until 70% confluence was reached. Cells received the addition of different concentrations (1, 2, and 10 μM) of the proteins and were incubated for an additional period of 48 h. The cytotoxicity of the proteins was assessed using the CellTiter-Glo Luminescent Cell Viability Assay (Promega) on a Victor 3 luminescent plate reader (PerkinElmer). These experiments were conducted in triplicate, and the results were expressed as the percentage (%) of cell viability ± the standard error.

### Confocal Microscopy in Caco-2 Cells

Caco-2 cells, derived from human colonic adenocarcinoma cancer cells, were used in this assay. Cells were maintained in Minimum Essential Medium (MEM) and seeded in eight-well μ-Slide Grid-500 high iBiTreat (80806, Ibidi) plates at a density of 60,000 cells/cm^2^. Media was changed every 2–3 days, and cells were grown for 21 days to form a tight epithelial monolayer. Cells were then incubated at a concentration of 10 μM of GFP-H6 and c-CPE-GFP-H6 in OptiPro SFM (Gibco) for 1 h. Untreated cells were used as a control. Then, protein was removed, and cells were washed with PBS and stained with Hoescht 33342 at 1 μg/mL (for labeling nuclei) and Wheat Germ Agglutinin – Alexa Fluor 555 (WGA 555, W32464, Invitrogen, ThermoFisher Scientific) at 6.66 μg/mL (for labeling cell membranes) in OptiPro SFM. Images were obtained using a Zeiss LSM 980 confocal microscope (Zeiss) equipped with a 63*x*/1.4 oil immersion objective lens. Excitation reached 553 nm for WGA 555, 488 nm for GFP, and 348 nm for Hoechst 33342. Emission was detected at 527–735 nm for WGA 555 (red), at 490–546 for GFP (green), and at 408–496 for Hoechst 33342 (blue). Emission detection settings were optimized to avoid cross-talk between channels. The WGA 555 detector operated at a gain voltage of 650 V, the GFP detector at 620 V and the Hoechst 33342 detector at 560–640 V. Images were acquired equally and processed equally in all cases (display adjustment for GFP was set at 24–200 in control, GFP-H6 and c-CPE-GFP-H6 images). Imaris Viewer 9.5.1 and ImageJ 1.53c were used for 2D data visualization and analysis. Spatial analysis shown in Figure 4C was obtained using Plot Profile tool from ImageJ, opening images as “colorized” to ensure measurement coincidence. For three-dimensional reconstructions, images were obtained at different *z*-axis levels (62 slices, 18.3 μm). Images were then visualized by sections (Figure 4B) and processed with Imaris 7.2.1 using masks (Figure 4D) for both blue and green channels.

### Permeability Assay in Caco-2 Cells

Caco-2 cells were used in this assay. Cells were maintained in Minimum Essential Medium (MEM) (Gibco) supplemented with 10% fetal bovine serum (Gibco) in a humidified atmosphere with 5% CO_2_ at 37 °C. For this assay, the cells were seeded in transwell inserts (1.12 cm^2^) with a pore size of 0.4 μm (60,000 cells/insert). The basolateral and apical compartments were filled with 2 mL of MEM medium supplemented with 10% FBS. The cells were allowed to grow for 21 days to form a tight epithelial monolayer. After this period, cell monolayer integrity was confirmed via transepithelial electrical resistance (TEER) and also by the decrease in the fluorescence intensity at 530 nm of Lucifer Yellow (λ_ex_ = 435 nm), a fluorophore that crosses the empty transwell and is used as a control of monolayer integrity. On day 21, the protein samples were added into 0.5 mL of the corresponding buffer and 1.5 mL of the same buffer was used to fill the basolateral well, at a concentration of 10 μM. After 1 h of incubation at 37 °C, the basolateral sample was collected and analyzed by fluorescence intensity (λ_ex_ = 488 nm) to determine the amount of protein that passed through the monolayer. When GFP-H6 was coincubated with c-CPE-FGF2-H6 nonfluorescent protein, this protein was incubated at 0.4 μM.

To confirm the lack of effect in Caco-2 cell viability, 20,000 cells were seeded in each well using a 96-well plate. After 48 h, proteins (GFP-H6, c-CPE-FGF2-H6, and c-CPE-FGF2-H6 + GFP-H6) were added at the same concentration of the permeability assay for 1 h. The cytotoxicity was measured as previously described for HeLa cells.

### Calibration Curve of Proteins in MEM Alpha Medium and Calculations of Permeability

The efficiency of different proteins in crossing the Caco-2 monolayer was determined by measuring the fluorescence in the basolateral chamber, and this value was used to calculate the protein concentration in nM. However, as proteins may have different fluorescence intensities in the medium, we first determined the specific fluorescence of each protein in MEM alpha medium without phenol red. For that, each protein was diluted in MEM medium to several known concentrations, and the fluorescence intensity of the diluted proteins was acquired at 512 nm (as done for pure GFP proteins), with an excitation wavelength (λ_ex_) of 488 nm and in a concentration range of 0.05–2 μM. With this standard curve established, the fluorescence measured in each well could be used to determine the protein concentration.

### Statistical Analysis

Data were processed using GraphPad Prism 9.4.0 to perform the analyses and represent numeric data. When required, the Shapiro–Wilk normality test was performed to determine whether parametric tests were to be performed. An outlier detection method was applied using the modified Z-score, using a threshold of 3.5. Statistical significance was determined using one-way ANOVA followed by Tukey’s post hoc test for multiple comparisons or two-way ANOVA followed by Bonferroni’s post hoc test. For the two-way ANOVA, a more conservative approach was employed by using Bonferroni’s correction to control the likelihood of Type I errors across multiple comparisons. Differences between groups were considered statistically significant at *p* ≤ 0.05, denoted by *. Further levels of significance were indicated as ** for *p* < 0.01 and *** for *p* < 0.001.

## Results and Discussion

To examine the performance of a recombinant c-CPE domain as an enhancer of epithelial permeability, we fused c-CPE to the amino terminus of a hexahistidine (H6)-tagged GFP, resulting in the fusion c-CPE-GFP-H6 (Figure 1A–C). GFP-H6 was kept as a convenient experimental control, complemented with R9-GFP-H6, a related three-domain fusion protein in which the amino terminus of GFP-H6 contained the polyarginine R9, a well-known cell penetrating peptide, instead of c-CPE. The folding of GFP in these constructs was predicted to be similar, with high confidence (Figure 1B and Supplementary Figure 1), and the electrostatic charge was particularly positive in the case of the largely cationic R9-GFP-H6 (Figure 1C and Supplementary Table 1). In these multidomain proteins, both c-CPE and R9 were predicted to be solvent-exposed (Figure 1B and Supplementary Figure 1), as was H6 in all constructs (Figure 1B,C). R9 was selected as a functional tag because, in contrast to c-CPE, this cell-penetrating peptide^31,32^ has been identified as a mediator of the transcellular route in the epithelial passage of proteins^31,33–35^ (Figure 1D, middle), as opposed to the alternative paracellular pathway in which c-CPE is involved (Figure 1D, bottom). This distinction provides a valuable reference for analytical comparison together with the presumable lack of penetration of GFP-H6 (Figure 1D, top).

**Figure 1:**
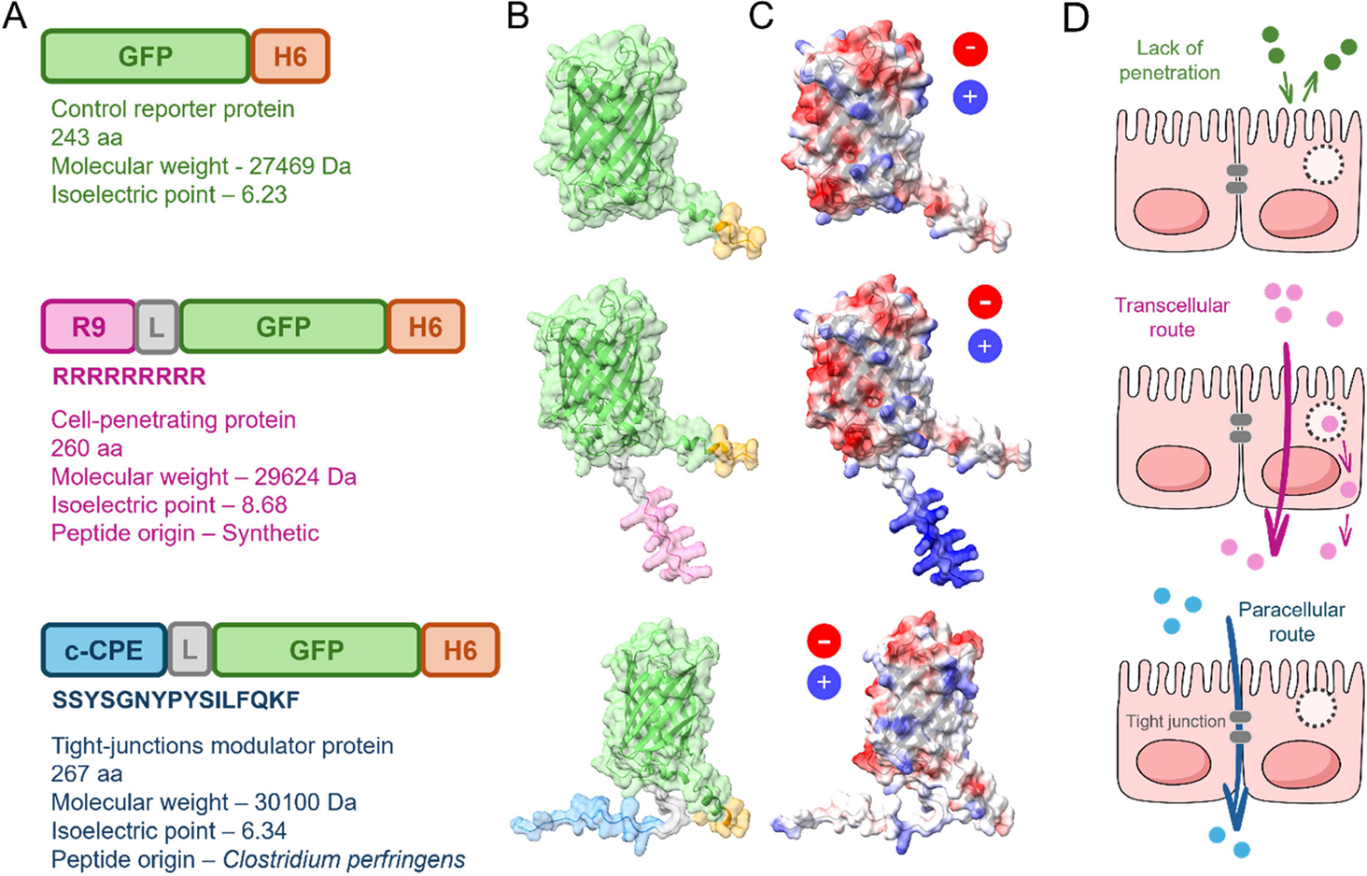
Multidomain proteins. (A) Modular organization of the recombinant proteins used in this study. The green fluorescent protein (GFP) is represented in green; H6, in orange, is a C-terminal hexahistidine tag; R9, in dark pink, is a cell-penetrating peptide; L, in gray, is a linker (GGSSRSS) that confers interdomain flexibility; and c-CPE, in blue, is the C-terminal region of C. perfringens enterotoxin. (B) Three-dimensional models of each multidomain protein generated by AlphaFold, with color legend as in panel A. (C) Three-dimensional models from B, colored according to their surface charge (red for anionic residues, blue for cationic residues, white for uncharged residues). (D) Schematic models of the epithelial permeability routes explored in this study. GFP-H6 should not penetrate cell-monolayers (top). The transcellular route relies on membrane activities (middle), whereas the paracellular route relies on the modulation of tight junctions (bottom).

The three fusion proteins were produced in Escherichia coli at reasonable yields and in the absence of signs of proteolytic instability (Figure 2A). As the GFP constructs are fluorescent (Figure 2B), the stability of the GFP β-barrel conformation was confirmed, a fact that supports the AlphaFold predictions regarding the solvent-exposure of the terminal peptide tags (Figure 1B). The estimation of the GFP-specific fluorescence of the model proteins (through measuring the fluorescence emission of equally concentrated protein solutions) revealed no decreases in the value of this parameter associated with the fusion of N-terminal peptides, either R9 or c-CPE (Figure 2B). In addition, circular dichroism shows that the secondary structure of all proteins was similar and highly overlapping between them (Figure 2C) and with the published spectra of native GFP.^36^ Thus, slight increases observed in the fluorescence of the N-terminal GFP-H6 fusions (Figure 2B) might be linked to subtle hydration changes revealed by DLS and thermal behavior (Figure 2D,E). In addition, these fusion proteins were all found in a soluble form, with a size compatible with those of GFP dimers (Figure 2D). These data indicated that the fused peptides had a negligible effect on the GFP structure, also supported by the absence of large size peaks in the DLS plots that, if occurring, might indicate aggregation (Figure 2D). Finally, in the same line of confirmation, the thermal stability of GFP-H6 was not impaired by the fusion of the N-terminal peptides at physiological temperatures, although an enhanced sensitivity to denaturing values (over 60 °C) was observed when aggregation started (Figure 2E). Altogether, these data confirm that the new fusion proteins were robust enough and suited for further analysis and that GFP fluorescence would be a convenient and stable marker of the localization of the protein.

**Figure 2:**
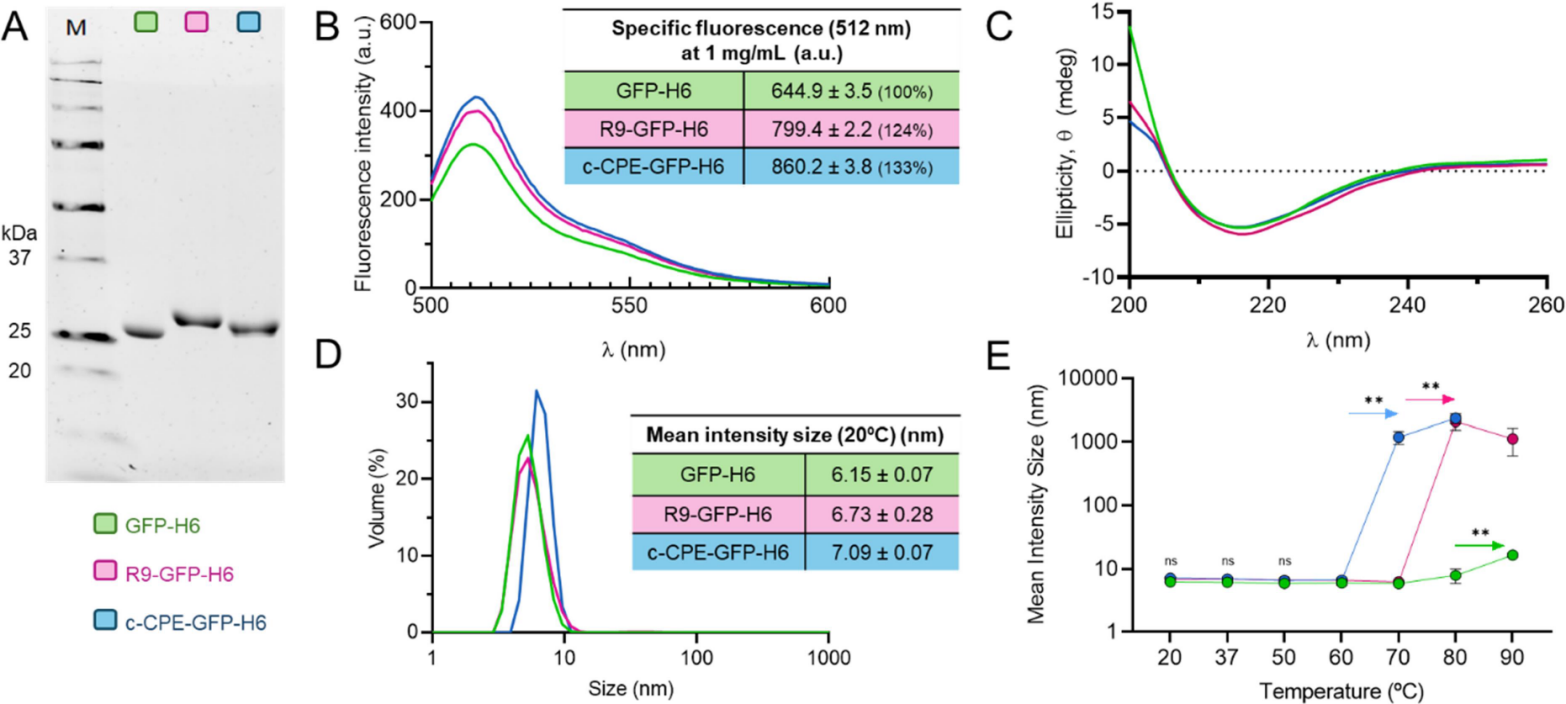
Physico-chemical properties of GFP-H6, R9-GFP-H6, and c-CPE-GFP-H6. (A) Comparative SDS-PAGE of each protein upon purification from cell extracts. Color legend (green for GFP-H6, dark pink for R9-GFP-H6, and blue for c-CPE-GFP-H6) is maintained hereafter. (B) Fluorescence intensity spectra upon excitation of GFP chromophore at 488 nm. The specific fluorescence peaks (measured at 512 nm, calculated for 1 mg/mL (a.u.)) are indicated in the table inset, along with the standard error of the mean (SEM). Inside brackets, the percentual specific fluorescence is shown, being 100% the one of GFP-H6. (C) Circular dichroism spectra for the three proteins. (D) Volume distribution (in %) of the hydrodynamic size of proteins at 20 °C, determined by DLS. In the inset, mean intensity data ± standard error of the mean (SEM) for each protein. (E) Thermal stability of proteins, as shown by the evolution of hydrodynamic size (in nm) upon temperature increase. Statistical differences between means are labeled as ** (*p* < 0.01). Nonsignificant (ns) differences were found in the 20–60 °C range.

At this stage, we tested the capability of these proteins to interact with cultured cells. In this context, R9-GFP-H6 but not the other GFP-containing proteins was found inside HeLa cells, with amounts increasing in a time-dependent manner (Figure 3A). This was expected, as R9, being a cell-penetrating peptide (CPP), is capable of using transcellular routes due to its capacity to efficiently penetrate cells,^37^ (Figure 1D). Then, we confirmed that all of the proteins were nontoxic at the tested doses (up to 10 μM) and remained so for at least 48 h of exposure (Figure 3B), allowing for further functional studies in cell culture interfaces. In this context, it must be noted that tight junction-active peptides are usually tested up to 100 μM, in absence of toxicity.^38^

**Figure 3:**
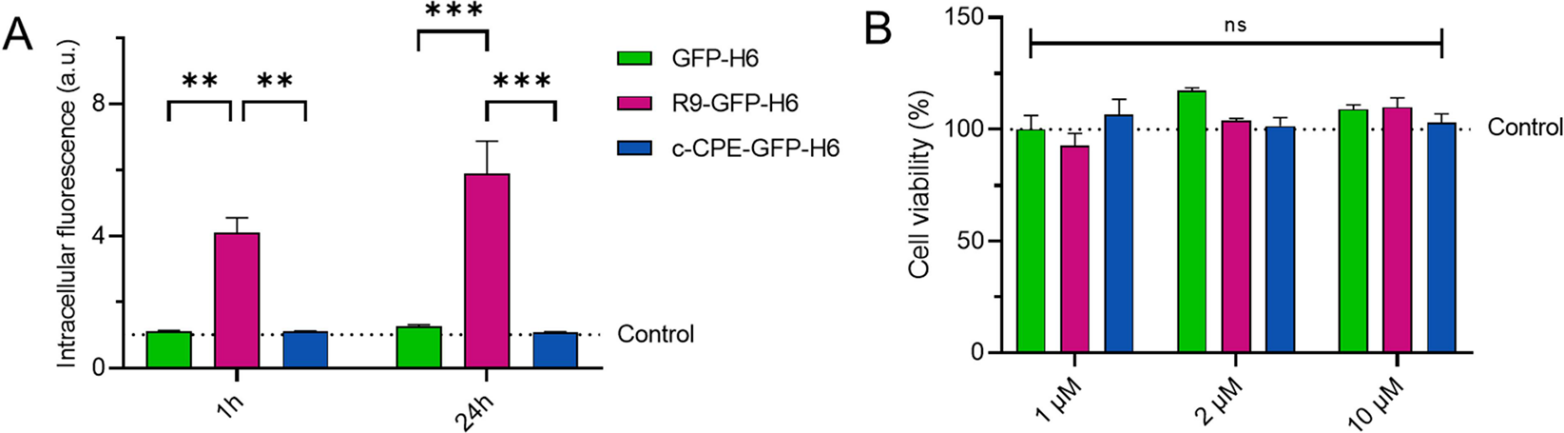
Cell penetrability and cytotoxicity of fluorescent fusion proteins. (A) Intracellular fluorescence (in arbitrary units, a.u.) emitted by HeLa cells exposed to 1 μM of either GFP-H6, R9-GFP-H6 or c-CPE-GFP-H6 for 1 or 24 h. Determination was done following a harsh trypsinization protocol to remove extracellular attached protein. One a.u. was set to the background fluorescence value of HeLa cells without protein exposure (control line). Thus, the values in the *Y*-axis indicate how many times more fluorescent the cells exposed to each treatment are. Statistical significances, established by two-way ANOVA followed by Bonferroni posthoc test, are indicated as ** *p* < 0.01 and *** *p* < 0.001. (B) Cell viability (%) upon exposure to either GFP-H6, R9-GFP-H6 or c-CPE-GFP-H6 at 1 μM, 2 μM or 10 μM, during 48 h. 100% represents the value for cells without protein exposure (control line). Statistical significance was assessed using a two-way ANOVA followed by the Bonferroni post hoc test.

At this point, we decided to examine the distribution of c-CPE-GFP-H6 upon exposure to cultured cells. For that, Caco-2 cells were cultured for 21 days to form a cell monolayer. Cells were incubated with c-CPE-GFP-H6 and GFP-H6 for 1h to determine their ability to bind cell monolayers. As observed, c-CPE-GFP-H6 but not GFP-H6, lacking c-CPE, was found in a pericellular location in the Caco-2 monolayer (Figure 4 A), in a location compatible with tight junctions, and in a punctuated and discontinuous pattern (Figure 4A, inset). It must be noted that c-CPE-GFP-H6 precisely labeled the cell–cell contact areas and not the apical cell membrane of the monolayer (Figure 4B), indicating the lack of a nonspecific binding with cell membranes, here stained in red. To further confirm that the localization between cells did not overlap with cell membranes, we evaluated a section of the monolayer and determined the intensity of each signal at each point using ImageJ. The intensity distribution reveals that the green signal corresponding to c-CPE-GFP-H6 is placed between the membrane signal but with no overlap (Figure 4C). The three-dimensional view (Figure 4D) confirms that c-CPE-GFP-H6 is located in the middle sections of the cell and not deposited on the surface, being observed around the cells and never inside the nuclei. These images match with those observed upon conventional labeling of TJ components.^39,40^ These results fully supported the TJ-binding abilities of the c-CPE-GFP-H6 construct and the strict dependence on its c-terminal peptide.

**Figure 4:**
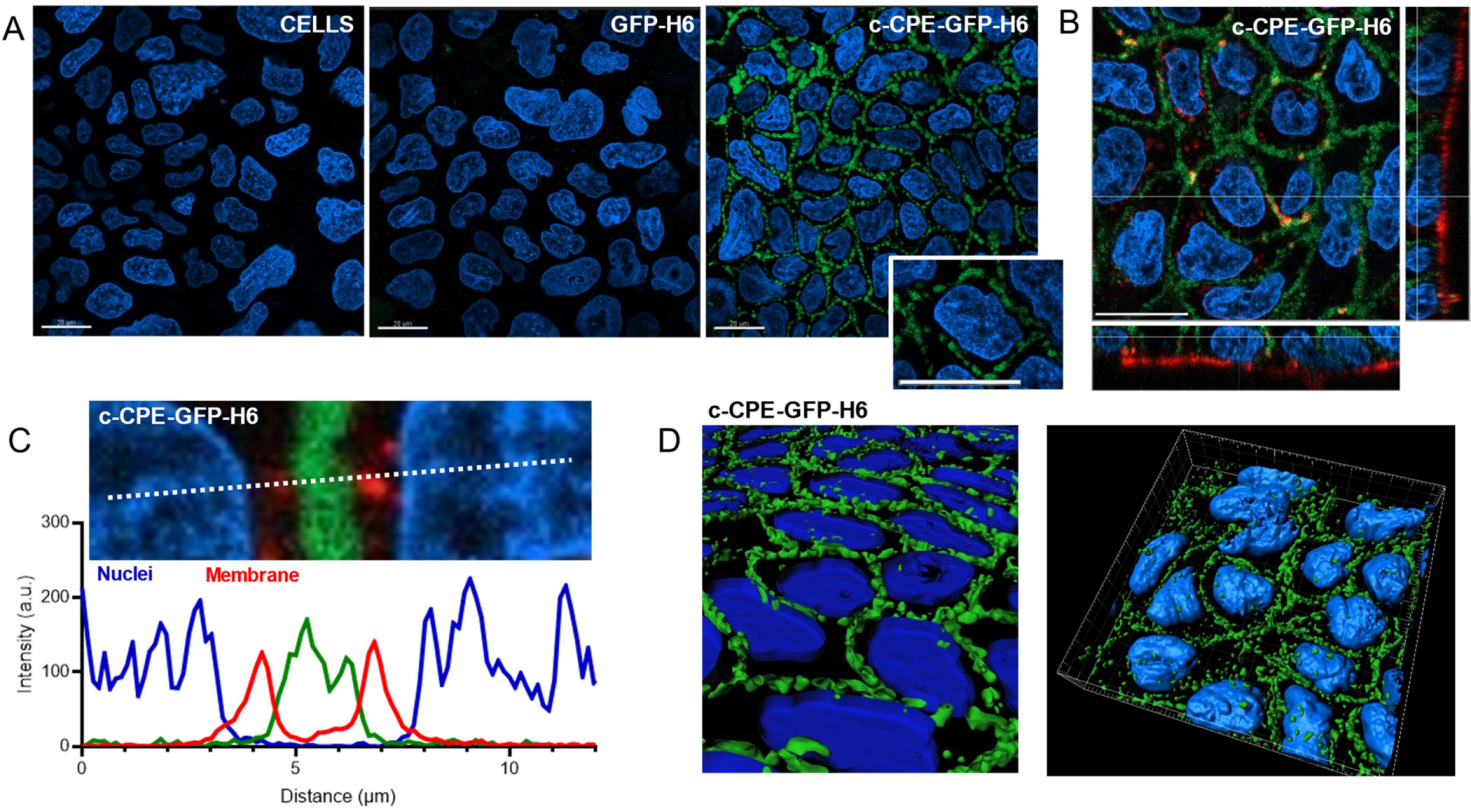
Confocal microscopy imaging demonstrating that c-CPE-GFP-H6 binds to tight junctions from a Caco-2 monolayer. (A) 2D images comparing fluorescence intensity and distribution on a GFP-H6-exposed or c-CPE-GFP-H6-exposed Caco-2 monolayer. Images were acquired and processed equally. Nonexposed cells (left) were used as a control. Hoescht 33342 (blue) was used to stain nuclei, and GFP fluorescence is shown in green. The inset highlights a cell surrounded by c-CPE-GFP-H6, in a discontinued punctuated pattern. The white bar indicates 20 μm. (B) Analysis of c-CPE-GFP-H6 distribution in the different z-planes. The Section tool from the Imaris Viewer was used to visualize the three-dimensional distribution of c-CPE-GFP-H6. Cell membranes were stained with WGA-555 (red). (C) Spatial analysis of the intensity of each fluorescence in a representative region of c-CPE-GFP-H6-exposed cells, indicating the distribution of c-CPE-GFP-H6 between neighboring-cells membranes. Intensity (au) was calculated with ImageJ. A discontinuous white line indicates the region of analysis. (D) Three-dimensional reconstructions of c-CPE-GFP-H6 using masks for both green (protein) and blue (nuclei) fluorescence. All images were acquired from cells grown for 21 days and proteins incubated for 1 h at 10 μM, which were removed before imaging.

Finally, we wanted to explore the role and performance of the recombinant c-CPE in permeabilizing tight junctions in a Caco-2 cell monolayer grown for 21 days (Figure 5A), a platform routinely used as a convenient model to screen epithelial permeability in a diverse range of biological contexts and tested agents.^41–46^ For studying this phenomenon, a correlation of fluorescence specifically determined in the experimental media and protein concentration was mathematically obtained (Figure 5B) to estimate protein amounts from the raw analytical data (fluorescence) obtained in the basolateral chamber. Sufficiently high protein concentrations (10 μM) were used to avoid sensitivity issues in the fluorescence detection, envisaging potentially limited penetrability. As expected (Figure 1D), the nonfunctionalized GFP-H6 control was unable to cross the monolayer (Figure 5C), while the addition of R9-GFP-H6 resulted in a significant increase of protein crossing beyond the cell layer. This fact, of course, must be attributed to the transcellular pathway reached by this protein (Figure 1D) resulting from its cell-penetrating activities (Figure 3A). Interestingly, c-CPE-GFP-H6 showed a tendency to cross the cell monolayer (Figure 5C), especially compared with the control GFP-H6. However, the amount of penetrated protein was not significantly different upon ANOVA analysis when compared to GFP-H6 (*p* = 0.58, Supplementary Table 2). The binding of c-CPE to the tight junctions (Figure 4), linked to their conformational relaxation, could be too tight to allow permeability of the same effector molecule and is therefore responsible for the moderate amounts of c-CPE-GFP-H6 found at the basolateral side of the trans-well system. To check this hypothesis, we investigated whether the permeability of GFP-H6, intrinsically unable to cross the Caco-2 monolayer, was promoted in trans by another c-CPE-containing protein, which should be nonfluorescent because of analytical needs. In this context, we generated c-CPE-FGF-2-H6 (Figure 5D), in which the fibroblast growth factor 2, irrelevant regarding tight junction performance but providing a control in the context of modular fusion proteins, could offer a trans-acting c-CPE domain to favor the penetration of GFP-H6. Like in the natural toxin,^24,47^ the c-CPE segment was solvent-exposed in c-CPE-FGF-2-H6, and therefore, it appeared suited for cell interactions (Figure 5D and Supplementary Figure 1). Indeed, a mixture of c-CPE-FGF-2-H6 and GFP-H6 at a low molar ratio (4:100) resulted in significantly higher amounts of GFP-H6 crossing the monolayer, comparable to those reached by R9-GFP-H6 (Figure 5C). To dismiss any proliferative effect of FGF-2 on Caco-2 cells, which might have altered the above data, a cell viability test was performed. This analysis confirmed, as expected, that under the experimental conditions, that is, 1 h exposure, the FGF-2 construct did not alter cell growth (Supplementary Figure 2).

**Figure 5:**
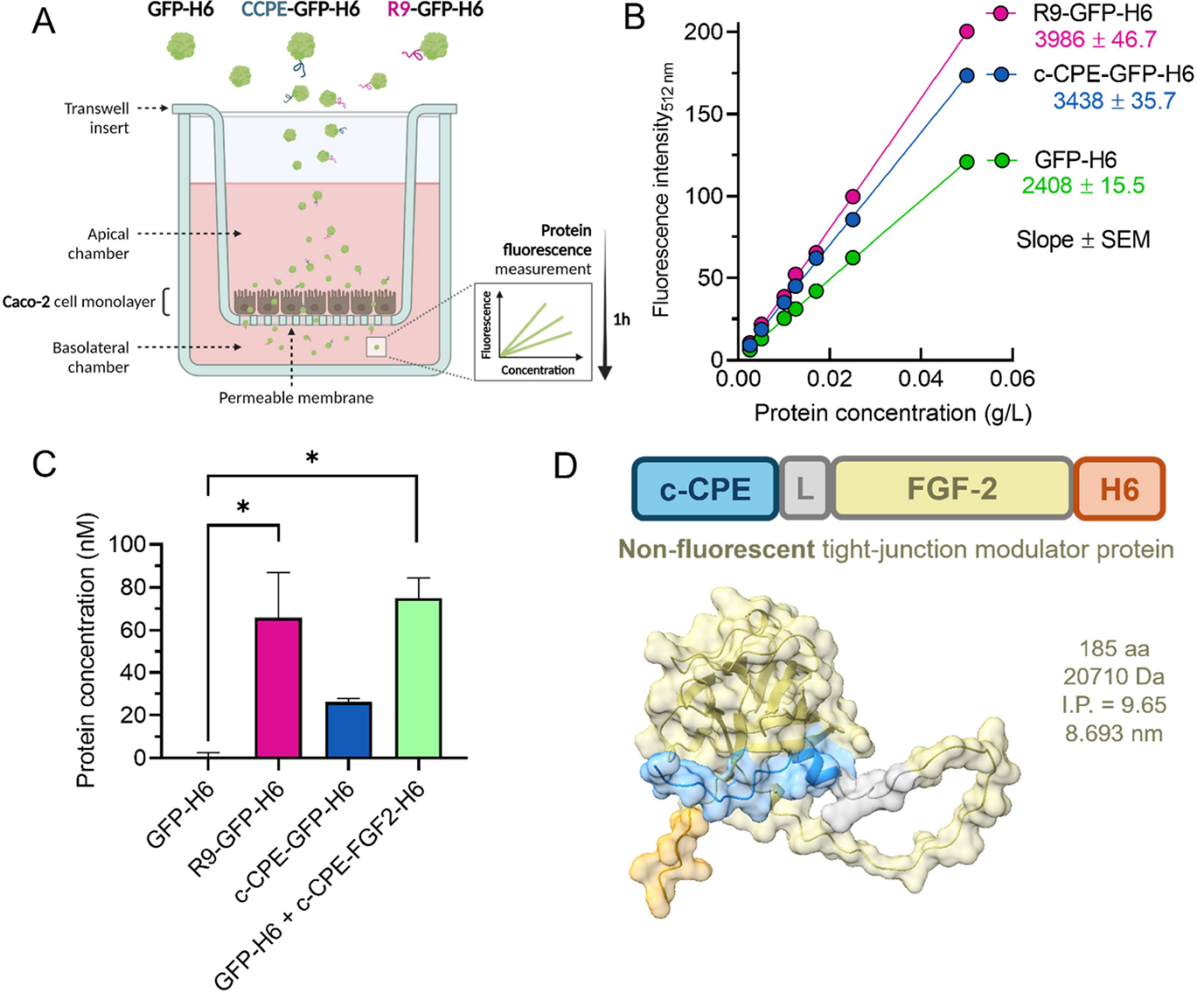
Protein penetration through a Caco-2 cell monolayer. (A) Schematic representation of the transwell-based Caco-2 cell as a model for epithelial permeability. (B) Fluorescence calibration curve of proteins in the presence of MEM alpha media without phenol red. Slopes for each protein curve, indicated in color, were used to infer protein concentration from fluorescence data. Slight discrepancies with relative data from Figure 2B might be due to the different composition and pH of the media where proteins were analyzed. (C) Protein concentration (in nM) in the basolateral chamber of the Caco-2-monolayer transwell. GFP-H6 was also coincubated with c-CPE-FGF2-H6 (at 0.04 fold molar ratio, 400 nM). Statistical significance was established by one-way ANOVA followed by Tukey posthoc test (*p < 0.05). Details of the statistical analysis can be found in Supplementary Table 2. (D) Modular disposition, 3D model and relevant physicochemical data of c-CPE-FGF2-H6, the nonfluorescent tight junction modulator.

## Discussion

In the context of transepithelial drug delivery, TJs play a critical role as adjustable biological barriers whose activity can be modulated through intervention over their components.^8,48^ c-CPE is a bacterial toxin segment with the ability to bind claudins, a component of TJs, and to promote changes in their permeability.^49^ The TJ binding activities of c-CPE have been explored, when used in recombinant form or as macromolecular complexes, as a diagnostic probe or marker in cancer.^50–52^ c-CPE, as a peptide, enhances epithelial permeability of both small molecules such as fluorescein or large proteins such as albumin,^24^ being an interesting agent in the formulation of drugs intended for local action. In this landscape, we wondered if a recombinant form of c-CPE, fused to a biologically functional protein (here GFP as model, Figure 1), could mediate its own passage through a Caco-2 monolayer (Figure 5A) commonly used as a reliable epithelial model.^41^ c-CPE-GFP-H6 showed only a residual, nonsignificant crossing of the layer, in contrast to the efficient penetration by R9-GFP-H6 (Figure 5C), empowered with a CPP that promotes the transcellular route (Figure 1). The fact that the c-CPE peptide acts in trans,^24^ per se, is not a satisfying explanation for the failure in the paracellular passage of c-CPE-GFP-H6, since the trans-activity should be feasible between homologous c-CPE-carrying molecules. Because of the important claudin-binding properties of c-CPE,^53,54^ it might be possible that c-CPE-bearing proteins have a tendency to attach to TJs, even persisting after their relaxation. The residual fluorescence detected in the transwell would then result from a small fraction of detached or apically saturated polypeptides. This hypothesis was fully supported by challenging the Caco-2 monolayer with a mixture of GFP-H6 (fluorescent, lacking claudin-binding agent) and c-CPE-FGF-2-H6 (nonfluorescent, displaying c-CPE), which resulted in the paracellular passage of GFP-H6 at levels comparable to those of R9-GFP-H6 (Figure 5C). Therefore, while c-CPE itself could potentially act as a driver of functional fusion proteins through epithelia, its claudin-binding properties (responsible for an enhanced permeability) appear to minimize its full potential when used in cis. Alternatively, the application of c-CPE in trans, even as part of a complex and large fusion protein, is highly effective in favoring the delivery of heterologous polypeptides that remain functional after the paracellular route. Importantly, the trans-activity of c-CPE over the epithelial model is observed at nontoxic concentrations (Figure 3B), which makes feasible the design of active and efficient drug formulations (including protein-based) in which c-CPE is intended as an effector but not as a self-driver. How the dosage of trans-acting c-CPE could be adjusted to appropriate levels for optimal drug penetrability is a matter of further exploration through in vivo functional analyses.

## Conclusions

c-CPE, as a component of recombinant proteins, has been described here as a nontoxic TJ binder and regulator, useful for promoting paracellular epithelial permeabilization. Paradoxically, while c-CPE allows the efficient transfer of c-CPE-lacking proteins, the domain is unable to allow the migration of self-containing fusion proteins, probably because of the high affinity of the peptide for TJ components (mainly claudin 4). Therefore, c-CPE in drug formulations for epithelial delivery must be considered only as a trans-acting agent but not as a cis-acting functional domain for the simultaneous TJ relaxation and self-driving (as fusion proteins or macromolecular drug complexes) through the paracellular space.

## Supplementary Material



## Notes

Net charge of the proteins and peptides used in the study based on the ionization states of their amino acid residues at pH 7.4, statistics of the permeability assays shown in Figure 4C, prediction confidence of the protein models obtained by AlphaFold, and Caco-2 viability (to discard toxicity) upon exposure to the proteins studied here (PDF)

## Abbreviations

c-CPE: the C-terminal domain of the C. perfringens enterotoxin
GFP: green fluorescent protein
FGF-2: fibroblast growth factor 2
H6: hexahistidine
R9: nonaarginine
TJ: tight junction
