# Supplementary material for: Trans-Mediated, Cis-Inhibited Paradoxal Activity of Clostridium perfringens Enterotoxin (c-CPE) in Modulating Epithelial Permeability
Source: Mol Pharm. 2025 Mar 11;22(4):1973–82. doi: 10.1021/acs.molpharmaceut.4c01205 (PMC12123672; doi:10.1021/acs.molpharmaceut.4c01205)
Supplement: Supplementary file 1 [file mp4c01205_si_001.pdf]

Supporting Information

Trans-mediated, cis-inhibited paradoxal activity of *Clostridium perfringens* enterotoxin (c-CPE) in modulating epithelial permeability

Julieta M. Sanchez [1,2,3,4 ‡], *Marianna T. P. Favaro* [1,2 ‡], *Hèctor López-Laguna* [1,2,5], *Eloi Parladé* [1,2,5], *Angela di Somma* [1,6] *Isolda Casanova* [2,7,8], *Ugutz Unzueta* [2,5,7,8], *Ramón Mangues* [2,7,8], *Esther Vazquez* [1,2,5], *Eric Voltà-Durán* [1,2,5*] *and Antonio Villaverde* [1,2,5*]

[1] Institut de Biotecnologia i de Biomedicina (IBB), Universitat Autònoma de Barcelona, Barcelona, Spain.

[2] Centro de Investigación Biomédica en Red de Bioingeniería, Biomateriales y Nanomedicina, Instituto de Salud Carlos III, Barcelona, Spain.

[3] Departamento de Química, Cátedra de Química Biológica, Facultad de Ciencias Exactas, Físicas y Naturales, ICTA, Universidad Nacional de Córdoba, Av. Vélez Sársfield 1611, Córdoba 5016, Argentina

[4] Instituto de Investigaciones Biológicas y Tecnológicas (IIByT), CONICET-Universidad Nacional de Córdoba, Córdoba 5016, Argentina

[5] Departament de Genètica i de Microbiologia, Universitat Autònoma de Barcelona, Barcelona, Spain.

[6] Department of Chemical Sciences, University of Naples "Federico II", Vicinale Cupa Cintia 26, Naples, 20126 Italy

[7] Institut de Recerca Sant Pau (IR SANT PAU), Barcelona, Spain

[8] Josep Carreras Leukaemia Research Institute (IJC), 08916 Badalona, Spain.

Supplementary Table 1. Net charge of the studied proteins and peptides based on the ionization states of their amino acid residues at pH 7.4.

	**Net charge (z-value)**	
**Protein**	**N-terminal peptide**	**Full protein**
GFP-H6	-	-8.177
R9-GFP-H6	+8.186	+1.786
c-CPE-GFP-H6	+0.209	-6.191

Supplementary Table 2: Statistical summary for permeability assays (Figure 4C).

**Tukey's multiple comparisons test**	**Mean Diff,**	**95.00% CI of diff,**	**Summary**	**Adjusted P Value**
**GFP-H6 vs R9-GFP-H6**	-65.38	-130.3 to -0.4731	*	0.0485
**GFP-H6 vs c-CPE-GFP-H6**	-25.88	-90.78 to 39.03	ns	0.5801
**GFP-H6 vs GFP-H6 + c-CPE-FGF2-H6**	-74.54	-139.4 to -9.640	*	0.0268
**R9-GFP-H6 vs c-CPE-GFP-H6**	39.50	-18.55 to 97.55	ns	0.1989
**R9-GFP-H6 vs GFP-H6 + c-CPE-FGF2-H6**	-9.167	-67.22 to 48.88	ns	0.9509
**c-CPE-GFP-H6 vs GFP-H6 + c-CPE-FGF2-H6**	-48.67	-106.7 to 9.385	ns	0.1006

*: $p \leq 0.05$

ns: non significant

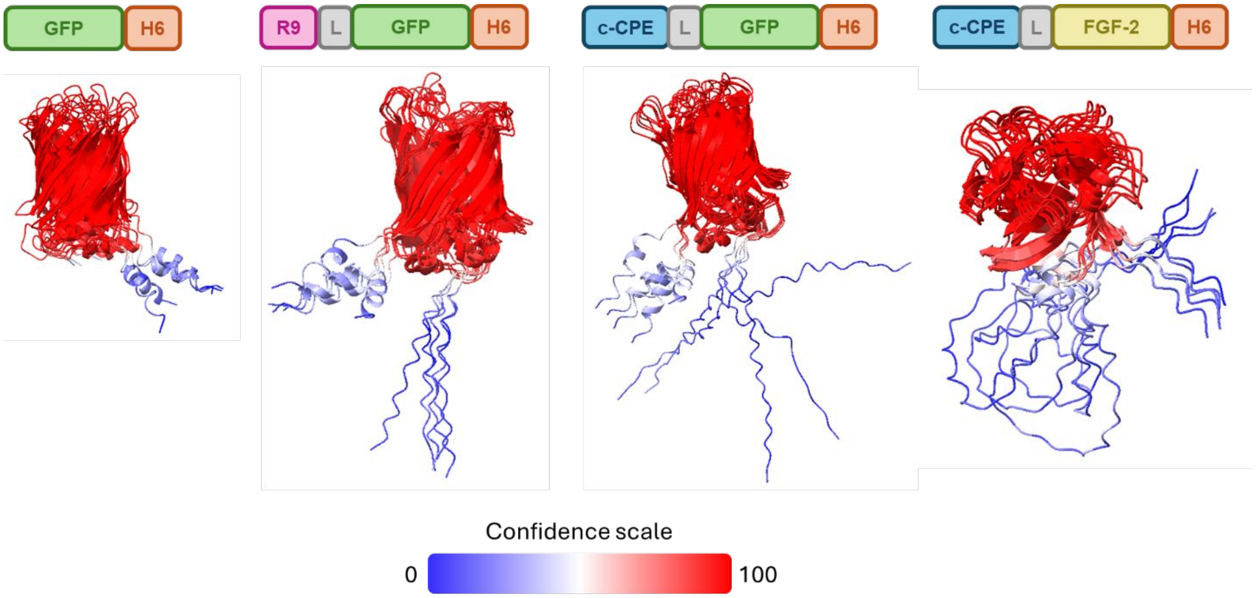

Supplementary Figure 1. Prediction confidence of models obtained by AlphaFold, in a blue-to-red (0-100) color scale. The 5 models obtained for each protein were superimposed, indicating high confidence and similarity in the scaffold (GFP, FGF-2) structure and the solvent exposure of both N-terminal and C-terminal domains.

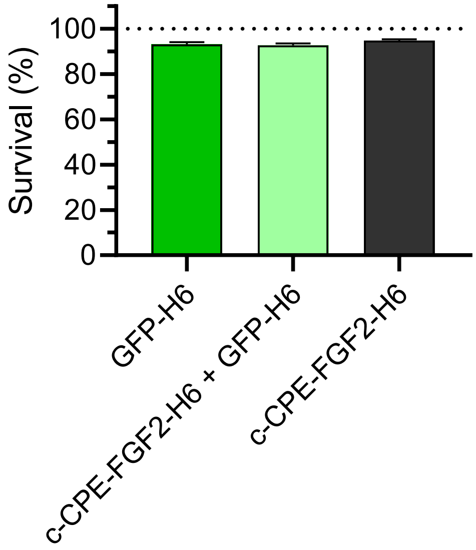

Supplementary Figure 2. Caco-2 viability (in %) upon exposure to either 10 µM GFP-H6, 0.4 µM c-CPE-FGF2-H6, and a combination of c-CPE-FGF2-H6/GFP-H6 at 0.4 µM and 10 µM respectively, during 1 h. 100 % represents the value of viability in cells without protein exposure (control line). Statistical significance was assessed using a one-way ANOVA followed by Tukey's post-hoc test.
